# Vascular Deformation Mapping of Abdominal Aortic Aneurysm

**DOI:** 10.3390/tomography7020017

**Published:** 2021-05-13

**Authors:** Drew J. Braet, Jonathan Eliason, Yunus Ahmed, Pieter A. J. van Bakel, Jiayang Zhong, Zhangxing Bian, Carlos Alberto Figueroa, Nicholas S. Burris

**Affiliations:** 1 Section of Vascular Surgery, Department of Surgery, University of Michigan, Ann Arbor, MI 48109, USA; djbraet@med.umich.edu (D.J.B.); jonaelia@med.umich.edu (J.E.); figueroc@med.umich.edu (C.A.F.); 2 Department of Cardiac Surgery, University of Michigan, Ann Arbor, MI 48109, USA; yuah@med.umich.edu (Y.A.); petrusv@med.umich.edu (P.A.J.v.B.); 3 Department of Radiology, University of Michigan, Ann Arbor, MI 48109, USA; jyzhong@umich.edu (J.Z.); zxbian@umich.edu (Z.B.); 4 Department of Biomedical Engineering, University of Michigan, Ann Arbor, MI 48109, USA

**Keywords:** abdominal aortic aneurysm, aneurysmal enlargement, vascular deformation mapping

## Abstract

Abdominal aortic aneurysm (AAA) is a complex disease that requires regular imaging surveillance to monitor for aneurysm stability. Current imaging surveillance techniques use maximum diameter, often assessed by computed tomography angiography (CTA), to assess risk of rupture and determine candidacy for operative repair. However, maximum diameter measurements can be variable, do not reliably predict rupture risk and future AAA growth, and may be an oversimplification of complex AAA anatomy. Vascular deformation mapping (VDM) is a recently described technique that uses deformable image registration to quantify three-dimensional changes in aortic wall geometry, which has been previously used to quantify three-dimensional (3D) growth in thoracic aortic aneurysms, but the feasibility of the VDM technique for measuring 3D growth in AAA has not yet been studied. Seven patients with infra-renal AAAs were identified and VDM was used to identify three-dimensional maps of AAA growth. In the present study, we demonstrate that VDM is able to successfully identify and quantify 3D growth (and the lack thereof) in AAAs that is not apparent from maximum diameter. Furthermore, VDM can be used to quantify growth of the excluded aneurysm sac after endovascular aneurysm repair (EVAR). VDM may be a useful adjunct for surgical planning and appears to be a sensitive modality for detecting regional growth of AAAs.

## 1. Introduction

Abdominal aortic aneurysm (AAA) is a leading cause of morbidity and mortality in the US, resulting in up to 3% of all deaths among men who are 65–85 years old [1]. Although AAAs are typically asymptomatic, aneurysm rupture carries up to a 65–90% risk of mortality [2,3]. The risk of AAA rupture can be difficult to accurately estimate but generally increases with increasing maximum aortic diameter [4,5,6,7]. Imaging surveillance plays a central role in the management of asymptomatic aneurysms and maximum diameter remains the gold standard metric used to assess indication for surgical repair. AAAs with a diameter of less than 5.5 cm have been shown to have a low risk of rupture and thus are recommended to undergo serial imaging surveillance, with surgical repair reserved for AAAs that are ≥5.5 cm in males and ≥5.0 cm in females [5,8].

There is growing recognition that maximum diameter alone may be inadequate for evaluation of AAA. A prior publication has suggested that maximal diameter and rupture risk have an inconsistent relationship [9]. Further complicating the assessment of AAA growth, measurement variability with diameter measurements is substantial, with up to one third of computed tomography angiography (CTA) images having ±0.5 cm of variability in maximum diameter [10,11]. Average aortic growth rate by diameter assessment is around 0.43 cm per year [12]. Measurement variability of this magnitude makes confident determination of interval growth, defined as the change in the maximum diameter between two CTA images, challenging. Considering that surgical criteria employ diameter-based growth rates and absolute sizes, inaccuracies in aortic measurements may lead to inappropriate interventions. Additional features of AAA morphology, such as tortuosity, curvature, proximal neck angle, terminal aortic angle, presence of intraluminal thrombus (ILT), and the complex shape of the aneurysm itself influence the wall stress and thus potentially AAA growth and rupture risk [13,14,15]. Maximal aortic diameter measurement fails to capture these factors, does not account for growth at submaximal locations and simplifies growth to a two-dimensional process (in the plane of the aortic cross-section), rather than a complex three-dimensional (3D) phenomenon.

Volumetric measurements are a promising adjunct to diameter measurements that have been studied for investigating AAA growth and rupture risk and allow for identification of morphologic changes that are not reflected in maximal diameter [16,17,18]. Some authors suggest that the need for surgical intervention is better correlated with AAA volume rather than maximum diameter [19]. Although area and volume measurements may be more sensitive when considering overall growth, they remain subject to manual measurement variability and are unable to assess growth in a localized fashion. Additionally, size criteria are based on historic measurements, which may harbor inaccuracies in themselves, further emphasizing the need for improved aortic growth measurements. Vascular deformation mapping (VDM) is an emerging technique that uses deformable image registration (DIR) to quantify localized changes in aortic growth using routine clinical CTA images [20]. VDM has been previously described as a tool to assess growth of the thoracic aorta, but applicability of this technique to AAA has not yet been assessed [20,21]. Although computational methods have been used to assess 3D growth of AAAs, to our knowledge no prior studies have described the use of DIR for quantifying growth in AAA [22,23,24].

The objective of this study was to investigate the feasibility of the VDM technique for 3D assessment of growth of AAA dimensions using routine clinical CTA data from patients undergoing imaging surveillance. Additionally, we sought to compare the results of the VDM 3D growth analysis with those of the routine clinical CTA assessments to better understand the utility of this novel technique.

## 2. Materials and Methods

### 2.1. Study Population

Patients with previously diagnosed infrarenal abdominal aortic aneurysms were identified through a review of electronic medical records (EMRs) as having a recent CTA examination between June 2020 and August of 2020. After identifying the patient’s most recent CTA, the EMRs were reviewed to determine the which patients had prior CTA scans. Patients were included if they had infra-renal AAAs and ≥2 available good quality abdominal aortic CTAs. Patients were excluded for the following reasons: suboptimal abdominal aortic enhancement (luminal contrast <200 HU), non-contrast CT scans, juxta-renal or thoracoabdominal aortic aneurysms, or poor image quality due to noise/artifact. This retrospective analysis was approved by the local institutional review board (HUM00133798) and informed consent was waived.

### 2.2. Image Segmentation

DICOM data for CTA scans were exported and segmentation of aortic blood volume (i.e., AAA lumen) was completed using thresholding in six contiguous regions starting from the sub-diaphragmatic abdominal aorta and ending around the level of aortic bifurcation. All segmentations were completed using Mimics 22.0 Software (Materialise NV, Leuven, Belgium). A threshold was chosen on a case-by-case basis to isolate contrast-enhanced blood pool from the surrounding soft tissues and organs. A separate segmentation isolating intraluminal thrombus was completed using a user-defined threshold in four contiguous regions of the intraluminal thrombus. A Boolean operation was then used to join the segmentation of the aortic blood volume and the intraluminal thrombus (ILT), with the objective of creating an aortic mask that encompassed the entire AAA to the boundary of the aortic wall. Segmentations were then manually adjusted to exclude any extra-vascular tissue.

### 2.3. Vascular Deformation Mapping

**Figure 1:**
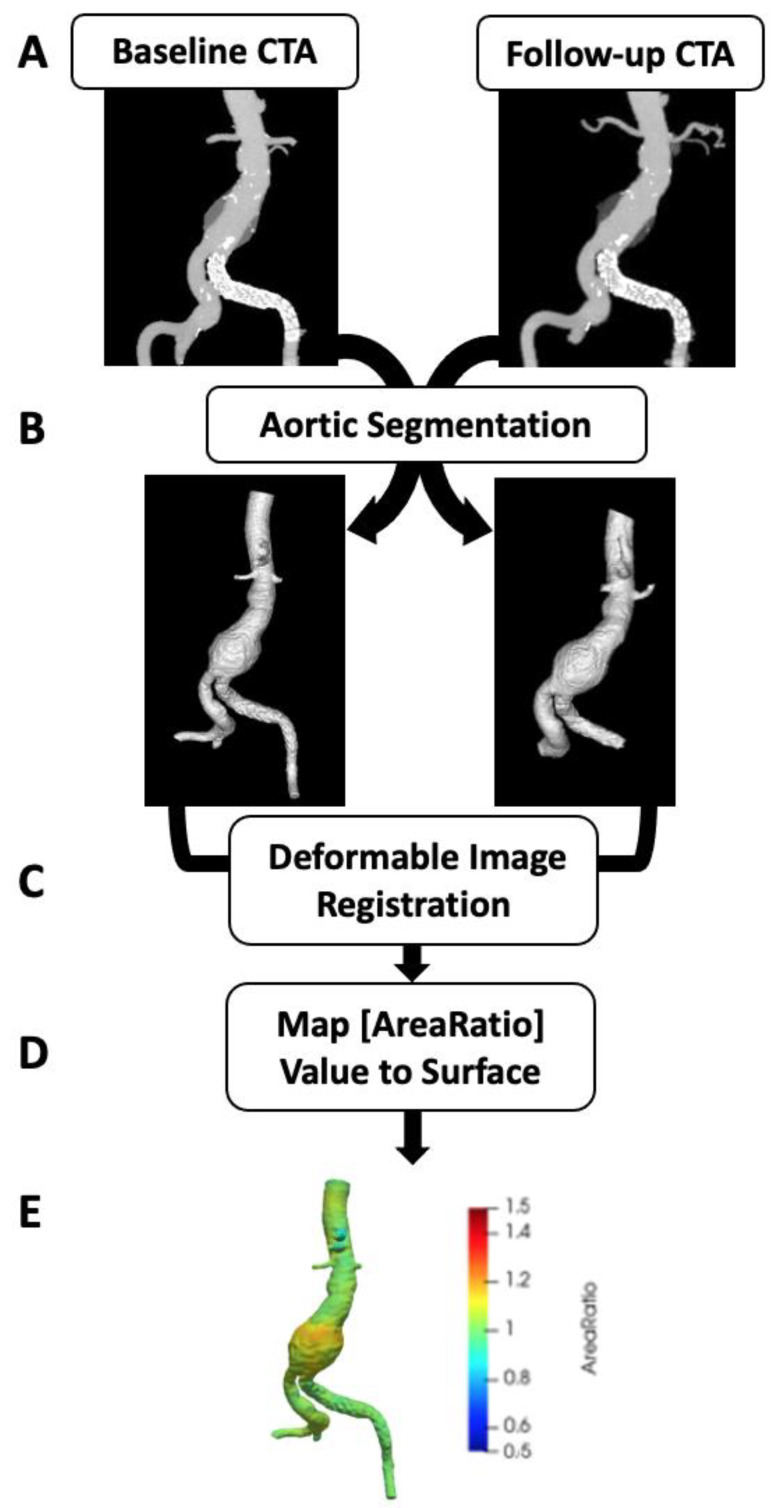
Vascular deformation mapping (VDM) workflow. Computed tomography angiography (CTA) images at two separate points in time are the source data for VDM (**A**) and undergo segmentation of to generate a mask of the AAA used to delimit the registration (**B**). Then multi-step registration is performed, first using rigid registration to align the 2 CTs followed by deformable registration to generate a deformation field, which is used to transform the baseline aortic mesh (**C**). The ratio of the surface area change (Area Ratio) at each triangular mesh element between baseline and follow-up CTAs is calculated (**D**) and the Area Ratio values are subsequently visualized by plotting values on the aortic surface using a colorized scale (**E**).

Vascular Deformation Mapping (VDM) involves multi-step image registration of aortic CTA studies, based on b-spline deformable registration techniques, to generate a deformation field which is subsequently used to quantify aortic growth in a 3D fashion. Such B-spline deformable image registration techniques have been shown to have submillimeter accuracy, and thus may yield significant improvements in measurement accuracy over other current aortic measurement techniques [25]. The steps involved in VDM analysis include: (1) segmentation of the abdominal aorta on CTA images from scans acquired at two different time points with the first time point considered the fixed image and the second time point considered the moving image; (2) image pre-processing including cropping and dilation of aortic masks by 3 voxels to ensure inclusion of the wall; (3) rigid registration (Euler) to approximately align the two CTA images (Elastix, Utrecht, Netherlands) [26]; (4) alignment of the aortic centerline using a highly regularized multi-image, multi-metric deformable registration which applies a penalty term to enforce rigid movement of voxels within the aortic segmentation but allows deformation of the peri-aortic voxels optimized rigid aortic registration [27]; (5) multi-resolution, multi-metric b-spline deformable image registration using mutual information with 10 mm grid spacing and a bending energy penalty of 20; (6) generate a polygonal mesh of the aortic surface (approximately 100,000–400,000 unique surface elements) at baseline (fixed) geometry; (7) translate mesh vertices of baseline model using the deformation field calculated in Step 5; (8) quantify deformation as the ratio of surface area change at each triangular mesh element (termed Area Ratio) with colorized visualization in Paraview (Kitware Inc., Clifton Park, NY, USA). The color scale is set so that aortic growth (Area Ratio > 1) is depicted by yellow-red colors and that aortic wall stability (Area Ratio ≈ 1) is depicted by green. A simplified workflow of the VDM technique is displayed in Figure 1. VDM outputs underwent a multi-step quality assurance (QA) protocol to ensure accuracy of results with basic steps including: (1) verify lack of segmentation errors, (2) inspection of the warped moving image after each sequential registration step to ensure appropriate transformation of the moving image towards to the fixed image target, and (3) inspection of a dual-channel image created using a gradient magnitude filter to enhance the aortic boundary, with fixed and warped moving images colored red and blue, respectively (i.e., area of image overlap display as purple). If a patient had multiple surveillance intervals (i.e., ≥1 CTAs) then VDM analysis was performed at each surveillance interval. However, only the longest surveillance interval for each AAA which passed QA assessments was included. Of note, any deformation detected at the end of vessels where segmentations were truncated (i.e., visceral & iliac arteries) were considered artifactual due to slight differences in segmentation extent.

### 2.4. Evaluation of Aortic Diameter and Volume

Clinical diameter measurements for each CT scan were obtained from clinical radiology reports and the maximal aortic diameter in the infrarenal segment was recorded for comparison with VDM results. Clinical maximum diameter measurements at our center are performed in a dedicated 3D analysis laboratory by experienced technicians using clinical analysis software (Vitrea, Vital Images, Toshiba, Tokyo, Japan) and centerline technique to obtain reformatted images in double-oblique plans. Maximum aortic diameter (including thrombus and the arterial wall) are then performed two directions perpendicular to the lumen centerline and these measurements are recorded in the clinical CT report. Volumetric measurements of the AAA were obtained using Mimics software. Aneurysm volume was determined by segmenting the AAA (lumen & ILT) from a level directly below the renal arteries down to the level of the aortic bifurcation. Growth was defined as “none/minimal” if clinical diameter increase was 0.0–0.3 cm, “moderate” if diameter change was 0.4–0.9 cm and “large” if diameter change was ≥1.0 cm; AAAs with “none/minimal” growth are considered “stable” given the known variability in aortic diameter measurements [21].

### 2.5. Statistics

Patient characteristics are reported as mean ± SD for continuous variables and frequencies for categorical variables. Pearson’s correlation coefficient was used to assess correlation between continuous variables. Statistical analyses were performed using Stata 14.0 (StataCorp LP, College Station, TX, USA).

## 3. Results

### 3.1. Patient Selection and VDM Technical Success

**Figure 2:**
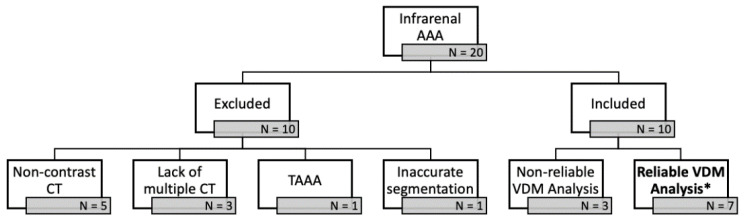
Twenty patients with previously diagnosed infrarenal abdominal aortic aneurysms (AAA) were identified through a review of electronic medical records. Of these patients 10 were excluded because of non-contrast imaging (*n* = 5), lacking multiple CT scans (*n* = 3), thoracoabdominal aneurysm (TAAA) (*n =* 1), and/or adjacent soft tissue structures (i.e., bowel/inferior vena cava) preventing accurate segmentation of the aortic wall (*n* = 1). Of the 10 patients that were included in the study, 7 had VDM analyses * that were deemed reliable after quality assessment steps.

Twenty patients with previously diagnosed infrarenal AAA were identified through a review of electronic medical records. Of these patients 10 were excluded because of non-contrast imaging (*n* = 5), lacking multiple CT scans (*n* = 3), thoracoabdominal aneurysm (*n* = 1), and/or adjacent soft tissue structures (i.e., bowel/inferior vena cava) preventing accurate segmentation of the aortic wall (*n* = 1). Among these 10 analyzed patients, there were a total of 26 CTAs and 22 surveillance intervals. Of the 10 patients that were included in the study, 7 had VDM analyses that were deemed reliable after QA steps (Figure 2). Among the 7 analyzed patients, VDM analysis of AAA growth was considered technically successful in 12/22 (54.5%) of surveillance intervals, but VDM analysis failed in 10/22 (45.5%) of evaluated intervals. The reasons for failure of the VDM analysis pipeline, as defined by the quality assessment (QA) protocol included: failure of adequate segmentation (*n* = 1), failure of rigid registration (*n* = 2), failure of deformable transformation (*n* = 2), and misregistration at the aortic wall after deformable registration (*n* = 5). Among the 10 failed VDM analysis, 5 (50.0%) were in case with ILT where failure was due to misregistration of the aortic wall boundary.

### 3.2. Patient Demographics

A majority of patients were male (6/7, 86%) and either former or current smokers (6/7, 86%). Average age was 65.1 ± 9.6 years (range: 56–82 years). One patient had a history of peripheral artery disease (PAD) and 4 patients had a history of coronary artery disease (CAD). PAD and CAD are defined as atherosclerotic narrowing or blockage of arteries in the leg or coronaries, respectively. One patient (AAA19) had a previous intervention, endovascular aortic aneurysm repair (EVAR). Five (AAA3, AAA6, AAA8, AAA9, and AAA11) patients eventually had interventions on their AAAs, all of which were treated via EVAR. Zero aneurysms ruptured in our cohort. Patient demographic and intervention information for the 7 patients included in this study are presented in Table 1.

### 3.3. Evaluation of Diameter and Volume

Interval time, change in volume (∆Volume), and change in maximum diameter (∆Dmax) can be seen in Table 2. Average baseline AAA diameter was 5.06 ± 1.17 cm and average baseline aneurysm volume was 11.96 ± 7.15 cm^3^. Three aneurysms (AAA3, AAA17, and AAA19) had minimal to no growth (0.0–0.3 cm) as assessed by maximum clinical diameter. Three aneurysms (AAA8, AAA9, and AAA11) had mild to moderate interval growth (∆Dmax 0.4–1.0 cm). One aneurysm (AAA6) had large interval growth (∆Dmax ≥ 1.0 cm) as assessed by maximum clinical diameter. Volumetric changes in AAA ranged from minimal (+1.79 cm^3^) to large (+72.01 cm^3^). One aneurysm (AAA19) demonstrated a slight interval decrease in volume (−5.09 cm^3^). There was moderate agreement between ∆Dmax and ∆Volume (R = 0.72, *p* = 0.07).

### 3.4. VDM Analysis

**Figure 3:**
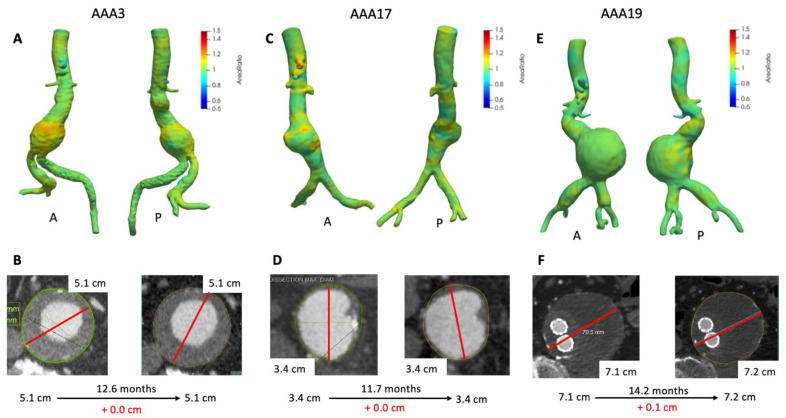
VDM analyses of AAA3, AAA17, and AAA19. VDM of AAA3 shows a moderate amount of growth anteriorly with some extending posteriorly (**A**), but maximum diameter was stable (**B**). VDM of AAA17 shows small punctate areas of growth anteriorly (**C**) but no growth by diameter assessment (**D**). AAA19 demonstrated no significant growth anteriorly, but there are two areas of subtle growth posteriorly (**E**), with maximum diameter change of +0.1 cm over 14 months (**F**). A = anterior projection, P = posterior projection.

Figure 3 depicts 3D growth maps for AAA3, AAA17, and AAA19 which had minimal to no growth based upon maximum diameter. Although AAA3 appears stable with respect to maximum clinical diameter (Figure 3B), there was a mild amount of growth seen on VDM (peak Area Ratio = 1.20). Most notably growth was localized to the anterior and superior aspects of the aneurysm sac at a level just above where clinical maximal diameter measurement was performed (Figure 3A). Additionally, there was a mild increase in volume, which further supports mild growth of this aneurysm (Table 2). Of note, this patient had a history of stent graft exclusion of the left common and internal iliac artery prior to CT scans of interest, which can be easily visualized on the VDM image. Patient AAA3 ended up getting an EVAR 1.5 years later after AAA grew to 5.6 cm in diameter. Conversely, VDM growth map for AAA17 demonstrated several small areas of deformation within the AAA (peak Area Ratio = 1.17) but the majority of the surface of the AAA demonstrated Area Ratio values ≈ 1, and both maximum clinical diameter and volumetric analyses were concordant with minimal to no growth over an 11.7 month interval (Figure 3C,D, Table 2). AAA19 had previously undergone an EVAR (4 years prior to imaging) with subsequent minimal increase in clinical diameter and a relatively stable volume (Figure 3E, Table 2). However, VDM growth assessment revealed two focal areas of mild deformation posteriorly (Figure 3F). This patient course was complicated by a possible small type II endoleak, which may correspond to lumbar vessels which course posteriorly near the regions of growth seen on VDM.

**Figure 4:**
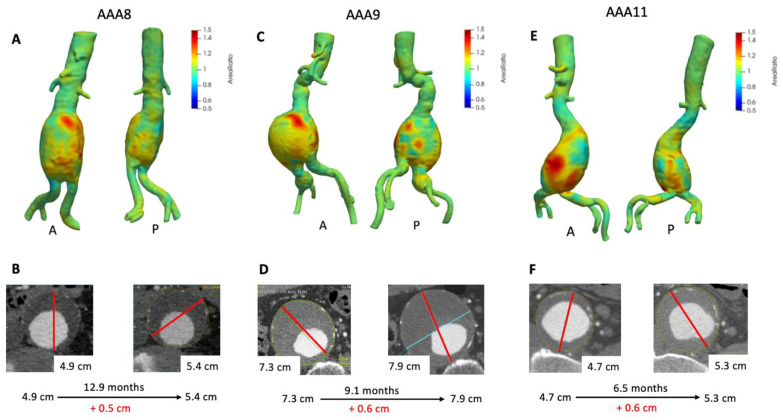
VDM analysis of AAA8, AAA9, and AAA11. VDM of AAA8 shows a significant amount of growth anteriorly and mild growth posteriorly (**A**), with interval increase of maximum diameter of 0.5 cm over 12.9 months (**B**). VDM of AAA9 shows an area of significant anterior growth and a few areas of growth posteriorly and laterally, and there was mild growth of a right common iliac artery aneurysm circumferentially (**C**) AAA9 demonstrated 0.6 cm of growth by diameter measurements over 9.1 months (**D**). VDM of AAA11 shows two areas of significant growth anteriorly and mild growth posteriorly (**E**), with interval increase of maximum diameter of 0.6 cm over 6.5 months (**F**). A = anterior projection, P = posterior projection.

Figure 4 depicts 3D growth maps for AAA8, AAA9, and AAA11; aneurysms with mild to moderate interval growth (0.4–1.0 cm) as assessed by clinical ∆Dmax. AAA8 increased about 0.5 cm in a one year period (Figure 4B) and VDM growth map clearly depicts diffuse circumferential deformation with a focal area of significant growth anteriorly (Figure 4A) with peak Area Ratio in this region of 1.58. In agreement with VDM and ∆Dmax, the aneurysm increased in volume by nearly 40 cm^3^ (Table 2). This patient underwent an EVAR about 1 month later given the interval growth. AAA9 increased in maximum diameter by 0.6 cm over 9.1 months (Figure 4D). VDM analysis depicts heterogeneous growth with focal areas of discrete growth both anteriorly (A) and posteriorly (P) (Figure 4C). The growth map demonstrates that there is diffuse deformation anteriorly but highlights a focal area of anterior growth with three small foci of growth posteriorly (peak Area Ratio 1.40). Volume measurements revealed that the aneurysm increased in volume by over 70 cm^3^ (Table 2). Additionally, a small amount of growth can be seen in the patient’s right common iliac aneurysm (Area Ratio = 1.3). This patient eventually underwent an EVAR with an iliac branch device to treat both their AAA and right common iliac artery aneurysm a few months later. Similarly, AAA11 was found to have an increase in maximum diameter of 0.6 cm (Figure 4F) and VDM revealed that the aneurysm has two focal areas of growth anteriorly and one posteriorly with some more diffuse areas of mild growth (Figure 4E) with peak Area Ratio of 1.48. In agreement with VDM and ∆Dmax, the aneurysm’s volume increased by nearly 37 cm^3^ over 6.5 months (Table 2). This patient underwent EVAR four months later given a maximum diameter of 5.5 cm.

**Figure 5:**
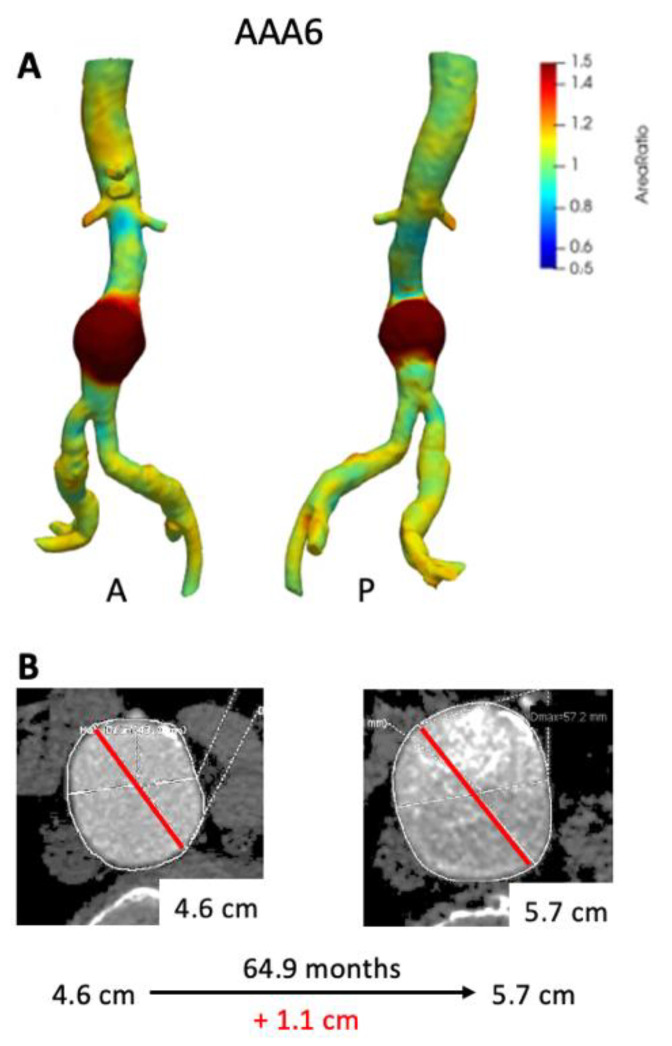
VDM analysis of AAA6 demonstrated a significant amount of growth circumferentially in the infrarenal abdominal aorta (**A**), and there was a corresponding increase in maximum diameter by 1.1 cm over 64.9 months (**B)**. A = anterior projection, P = posterior projection.

Figure 5 depicts growth maps for AAA patients that had large interval growth (>1.0 cm) based on ∆Dmax. AAA6 demonstrated a slow growth pattern over a 6-year period as exhibited by maximum clinical diameter (Figure 5B). VDM demonstrates fairly diffuse and circumferential growth of the AAA with peak Area Ratio of 2.01 with corresponding increase in aneurysm volume of over 40 cm^3^ (Figure 5A, Table 2). Of note, lower intensity growth (Area Ratio 1.2–1.4) was noted in the common iliac arteries and suprarenal abdominal aorta in patient AAA6, highlighting the diffuse nature of growth. This patient ended up undergoing EVAR several weeks after the last CT scan given that Dmax was above surgical threshold size.

## 4. Discussion

A primary rationale for the use of maximum diameter measurements to assess aortic growth progression and patient risk is based on the Law of Laplace, which states that the wall stress is directly proportional to the vessel radius and transmural blood pressure and inversely proportional to the vessel wall thickness. However, this law is greatly oversimplified relative to the many factors present in AAA and may be unreliable for accurately estimating tensile wall stress and predicting AAA growth for many reasons. First, the aorta is not a simple cylinder and does not have a single radius of curvature. Rather, AAAs have a complex and varying shape with both major and minor wall curvatures and variable vessel thickness [28,29]. This is further compounded by the presence or absence of intraluminal thrombus (ILT). The role of ILT in AAA remains unclear with some authors suggesting a protective mechanism [30,31] and others suggesting a deleterious role [32,33]. Nonetheless, the variable amount of ILT in AAAs creates further complexity, which is not addressed by maximal diameter. Moreover, maximum diameter may be inaccurate in predicating growth patterns and overall rupture risk. Among studies of ruptured AAAs, authors have demonstrated that 7.4% have a maximum diameter below 6 cm and 7.5% are below 5 cm [34,35]. Additionally, AAAs display a wide variety of growth ranging from completely stable to rapid growth (>1 cm/year). While small aneurysms typically display slow linear growth, up to 30% of such AAAs display non-linear, intermittent, and rapid growth behavior [13,36].

Volume measurements have been proposed as a promising adjunct to diameter measurements. Kauffmann et al., investigated the ability of a semi-automated segmentation combined with 3D–3D registration and found that 14% of patients had discordance between volumetric and diameter changes during follow-up [17]. These authors conclude that AAA volume was a more sensitive means to detect AAA growth than maximum diameter [20]. Furthermore, it has been demonstrated that a portion (2–18%) of patients who have increased aortic volume do not display corresponding axial or orthogonal diameter changes [16,18]. AAAs presenting rapid volume increase had a tenfold risk of undergoing surgery, while the risk was threefold for rapid maximum diameter increase [16]. While the advantages of volumetric assessment techniques for AAA are compelling, a remaining limitation of such measurements is that they do not provide any information as to the location of growth, but rather often only a global assessment of aneurysm dimensions.

The VDM technique offers unique advantages of understanding AAA behavior via a three-dimensional assessment of growth. First and foremost, growth can be visualized along the entire length of the aorta and around its circumference, whereas aortic diameter measurements are limited to a longitudinal and radial position along the aorta. Because VDM utilizes 3D image data, this technique avoids the 2D plane dependence of diameter measurements, a known case of diameter measurement variability. Moreover, the VDM technique uses non-rigid image registration techniques that can align CTA images with a precision in the range of 0.5–1.0 mm, far greater accuracy than can be expected from manual diameter measurements [25]. VDM poses an advantage over maximum diameter in that both localized and global growth can be assessed. The underlying molecular and biomechanical processes that drive AAA growth demonstrate significant heterogeneity [20,21]. Thus, the availability of a technique such as VDM that can assess growth in a localized fashion promises to advance our understanding of the relationship between growth and the underlying wall pathology. An additional advantage of a 3D growth assessment is that wall deformation can be assigned vectors of the magnitude and directionality of growth [37]. Lastly, VDM yields a more comprehensive assessment of growth than diameter measurements, allowing for identification of regions of slow, eccentric, and submaximal growth that may be nearly impossible to detect by diameter measurements.

In addition to demonstrating the feasibility of VDM for assessment of AAA growth, this study allows us to gain preliminary insights into the agreement between AAA growth assessments by diameter, volume, and 3D growth (VDM/Area Ratio). While some cases with minimal growth demonstrated concordant growth diameter and volume assessments (e.g., AAA17), others showed mild growth by volume and VDM, which was not appreciated by diameter measurements (e.g., AAA3). This observation is not surprising considering that volumetric assessment techniques have been reported to be more sensitive than diameter measurements [19]. Among cases with moderate and large growth we generally noted concordance between the various assessment techniques in terms of the presence of growth; however, VDM results clearly demonstrate that despite similar degrees of growth by diameter and volume change (e.g., AAA8, AAA9, and AAA11) the distribution and local intensity of growth can be quite variable. While the clinical significance of such difference in growth patterns remains unclear, we believe that the more detailed and accurate assessment of localized aortic growth afforded by VDM could improve our ability to predict future growth and adverse events in a fashion that has not been achievable with diameter measurements.

While only one patient in this small cohort has undergone prior endovascular repair, we were able to demonstrate that VDM is a feasible technique in the post-EVAR setting. AAA19 demonstrated a slowly growing aneurysm sac after EVAR, raising clinical concern of possible type II endoleak, which occurs when blood flows into the excluded aneurysm sac from aortic branches. Given the slow growth and location along the posterior wall, a lumbar artery would be a reasonable culprit vessel. The VDM growth map identifies two areas on the posterior surface of the aneurysm with focal growth which could correspond to anatomic location the lumbar arteries. While further study is needed, this result suggests that VDM yield useful information in the assessment of occult endoleaks. Further, while the precise effects of a metallic endograft on image registration accuracy remain uncertain, it is reasonable to suggest that high-contrast metallic stent struts may actually improve accuracy of registration algorithms.

This feasibility study is an important step in understanding the potential applications of VDM for evaluating AAAs. However, our study has several key limitations. First, this technique relies on segmentation of the AAA. While the contrast-enhanced aortic lumen is easy to segment with simple thresholding, segmentation of ILT is much more challenging. Boundaries of aneurysm wall and ILT (typically 50 to 100 Hounsfield Units) can be difficult to distinguish from adjacent soft tissue structures such as the bowel, peri-aortic fat, and unenhanced vessels (typically −100 to 100 Hounsfield Units). Thus, the VDM technique currently requires extensive and careful manual segmentation of the ILT to achieve accurate separation from adjacent structures. Second, this low contrast gradient between ILT and adjacent structures can significantly lessen registration accuracy, and misregistration at the aneurysm wall was deemed the cause of VDM analysis failure in approximately 22.7% (5/22) of intervals. We believe that the significantly higher rate of ILT in AAA is a main contributor to the higher VDM analysis failure rate in AAA (46%) versus in thoracic aneurysm (10%). Third, the VDM method requires significant technical expertise and is thus not currently suited for use in routine clinical practice. Fourth, our study involved only a small, heterogeneous sample of the overall AAA population at our center, and we did not explicitly aim to assess patient outcomes in this feasibility analysis. Lastly, the reproducibility of this technique was not assessed.

Future directions with the VDM technique will be threefold, with efforts focused on improving the technical aspects of VDM, further evaluating VDM in other clinical scenarios (e.g., repaired AAA, juxta-renal aneurysms, thoracoabdominal aneurysms, and pre-rupture states) and understanding the role of 3D growth patterns in predicting clinically meaningful outcomes such as growth trajectories, surgical outcomes, or adverse events. An important next step relies on systematic validation of the accuracy and consistency of VDM analysis. Additionally, technical developments are needed to improve AAA segmentation. Deep learning-based techniques have been described for accurate AAA segmentation and may be able to significantly lessen the need for manual involvement; specifically, improved segmentation methods for cases with ILT are needed. Furthermore, technical refinements to our multi-step registration procedures will be investigated to improve registration accuracy at the aortic wall in cases with ILT. Future clinical work will focus on feasibility and validation of VDM in the post intervention setting with a focus on the utility of VDM to evaluate the effect of endoleaks on excluded aortic sac dimensions after EVAR. Additionally, we would like to focus on the role of VDM in evaluating pre-rupture and rupture states, as this technique may be able to yield unique and novel information about specific 3D growth patterns that precede aneurysm rupture. Lastly, VDM may be a valuable research tool for study of aneurysm pathophysiology given its high degree of sensitivity of changes in aortic dimension, and if further validated and refined, VDM could potentially serve as an imaging biomarker to assess for longitudinal growth to judge the effectiveness of surgical or novel pharmacologic treatments.

## 5. Conclusions

We have demonstrated that VDM is a feasible technique to measure changes in the size of infra-renal AAAs using routine CTA data acquired in patients undergoing routine imaging surveillance. Additionally, we have shown that while growth assessments are largely concordant at large growth magnitudes, there are significant discrepancies between growth assessment methods at minimal to moderate degrees of growth, emphasizing the potential additive value of a 3D growth assessment technique that utilizes the full high-resolution CTA dataset and avoids the variability in measurement plane. VDM may be a useful adjunct for pre-surgical imaging surveillance and planning and yields both a quantitative measurement of localized changes in aortic surface area and a qualitative assessment of the unique growth patterns in AAA in a manner that is not achievable by existing techniques.

## Figures and Tables

**Table 1:** Patient Demographics.

Patient ID	Age *	Sex	Smoker *	PAD	CAD	Previous Intervention	Subsequent Intervention	Rupture
AAA3	73	Male	Current	Yes	Yes	No	Yes (EVAR)	No
AAA6	82	Male	Never	No	Yes	No	No	No
AAA8	80	Male	Former	No	No	No	Yes (EVAR)	No
AAA9	69	Male	Former	No	Yes	No	Yes (EVAR)	No
AAA11	56	Male	Current	No	No	No	Yes (EVAR)	No
AAA17	76	Female	Former	No	No	No	Yes (EVAR)	No
AAA19	78	Male	Former	No	Yes	Yes (EVAR)	No	No
Average	72.57							
SD	8.60							

Abdominal Aortic Aneurysm (AAA), Peripheral Artery Disease (PAD), Coronary Artery Disease (CAD), * at time of second CT scan, Endovascular Aortic Aneurysm Repair (EVAR).

**Table 2:** Clinical measurements of diameters and volume over time.

Patient ID	Dmax CT1 (cm)	Volume CT1 (cm^3^)	Dmax CT2 (cm)	Volume CT2 (cm^3^)	∆ Time (Months)	∆ Dmax (cm)	∆ Volume (cm^3^)
AAA3	5.1	124.15	5.1	136.02	12.6	0.0	11.87
AAA6	4.6	58.13	5.7	99.81	64.9	1.1	41.68
AAA8	4.9	113.41	5.4	151.26	12.9	0.6	37.85
AAA9	7.3	263.42	7.9	335.43	9.1	0.6	72.01
AAA11	4.8	103.18	5.2	139.95	6.5	0.4	36.77
AAA17	3.4	43.70	3.4	45.49	11.7	0.0	1.79
AAA19	7.1	243.48	7.2	238.39	14.2	0.1	−5.09
Average	5.06	11.96	5.51	14.69			
SD	1.17	7.15	1.33	9.04			

Dmax = maximum diameter based on computed tomography (CT) scans (numbered 1 and 2) SD = standard deviation.

## Data Availability

The data presented in this study are available on request from the corresponding author. The data are not publicly available due to use of protected personal health information.
